# Mineral Composition of Three Popular Wild Mushrooms from Poland

**DOI:** 10.3390/molecules25163588

**Published:** 2020-08-06

**Authors:** Michalina Gałgowska, Renata Pietrzak-Fiećko

**Affiliations:** 1 Department of Meat Technology and Chemistry, Faculty of Food Sciences, University of Warmia and Mazury in Olsztyn, Cieszyński 1 Sq, 10719 Olsztyn, Poland; michalina.galgowska@uwm.edu.pl; 2 Department of Commodities and Food Analysis, Faculty of Food Sciences, University of Warmia and Mazury in Olsztyn, Cieszyński 1 Sq, 10-726 Olsztyn, Poland

**Keywords:** edible mushrooms, *Boletus edulis*, *Boletus badius*, *Cantharellus cibarius*, minerals, microelements, macroelements

## Abstract

The region of Warmia and Mazury is characterized by the special diversity and richness of its natural environment, including large forest complexes, where wild mushrooms are commonly collected and consumed. This study aimed to examine the differences in mineral content (calcium, magnesium, sodium, potassium, iron, zinc, copper, manganese) of three species of mushrooms collected in north-eastern Poland. The research material consisted of dried samples of king bolete (*Boletus edulis*), bay bolete (*Boletus badius*), and chanterelle (*Cantharellus cibarius*) collected in the region of Warmia and Mazury. The content of the above-mentioned elements in mushroom fruit bodies was determined using the flame atomic absorption spectrometry (acetylene-air flame) and the emission technique (acetylene-air flame) for sodium and potassium. For the majority of micro- and macroelements, the studies confirmed the presence of significant differences in their content, depending on the species of fungi. The studied mushrooms cover a significant percentage of daily demand for many of the minerals. This concerns mainly copper, zinc, and potassium, although none of the species was a good source of calcium and sodium. Among the analyzed mushrooms, chanterelle is the best source of most minerals.

## 1. Introduction

The province of Warmia and Mazury is one of the least degraded areas in Poland, taking into account the natural environment. The whole province is located within the so-called “green lungs”, which covers the cleanest regions of the country. Forests are the natural wealth of the Warmia and Mazury region. The properties of woods (appropriate substrate, age of stands) favor the occurrence of undergrowth, e.g., mushrooms. According to the data from the Statistical Yearbook of Forestry, in 2018, the purchase of mushrooms amounted to 122 tons, which ranked the region 6th place in the country in terms of the amount of fungal raw material obtained [1,2]. The most valued mushrooms include *Boletus edulis, Boletus badius,* and *Cantharellus cibarius*.

*Boletus edulis* (King Bolete) is considered one of the most appreciated species of wild edible mushrooms and is a popular food throughout the world [3,4]. It is very popular mainly because of its aroma, texture, and the presence of nutrients as well as biologically active compounds, which determine its nutritional and medicinal properties [5,6,7]. The fruiting bodies of the bay boletus (*Boletus badius*) are tasty, which makes them widely used in gastronomy. Furthermore, they contain many valuable substances, such as antioxidants (anisole, BHT toluene, tocopherol) and rare metals (manganese, zinc, selenium). Chanterelles (*Cantharellus cibarius*) are a desirable trade item because of their attractive taste, durability during transport and storage, and the fact that the sporocarps are rarely affected by insect larvae. In addition, this mushroom is rich in natural vitamin C and contains high levels of potassium and vitamin D [8].

Wild mushrooms are commonly collected and consumed in Asia, Mexico, and Central–Eastern Europe. They are treated as an important food product, which is valued for its taste, nutritional value, and biological activity (antitumor, anticancer, anti-atherosclerosis and thrombosis inhibition, anti-inflammatory, hepatoprotective, and antihypertensive) [9,10,11,12,13,14,15,16]. Mushrooms are a good source of minerals, vitamins, potassium, dietary fiber, water, carbohydrates, and are low in lipids and sodium [17,18,19,20]. They are known to uptake and accumulate different chemical compounds [21,22,23,24,25], nevertheless the efficiency of this process may depend on various factors [19,26].

Currently, knowledge of the mineral content in edible mushrooms is relatively extensive. In many countries (South Africa, Turkey, Greece, Serbia, China, and others), numerous studies have been conducted on the mineral composition of various species of fungi to more accurately estimate their nutritional and health value and the mechanism of accumulation of individual components [22,27,28,29,30,31,32]. In Poland, large-scale analyses have been conducted, among others, by Falandysz, whose numerous reports concern a wide spectrum of elements determined in many species of mushrooms collected in various sites in Poland and around the world [33,34,35,36]. Siwulski, Mleczek, and Rzymski have also carried out numerous studies on cultivated and wild mushrooms from non-contaminated and contaminated areas [26,37,38,39,40,41].

Mushrooms owe their ability to accumulate micro- and macroelements to the specific structure of mycelium: the exposed surface of vegetative cells and large hyphae surfaces [42]. They are able to store minerals in large quantities even exceeding concentrations found in the medium in which they have grown [43,44]. The uptake of elements considered physiologically essential to mushrooms (K, P, Mg, Mn, Cu, Ca, Na, Zn) by mycelia and their deposition in fruiting bodies is species dependent [45]. Therefore, unlike vascular plants, mushrooms are able to accumulate high concentrations of minerals, even when growing in soils with low metal contents [35]. In this way, they become a particular rich source of minerals. In Kalač’s opinion, the levels of elements in wild mushrooms significantly increase with the increasing age of mycelium and extended time between the fructifications [46]. Variability in the chemical composition of mushrooms within a species is greater than that of plants. Since each individual fruit body can result from the crossbreeding of different hyphae and so presents a distinct genotype, the mineral contents in a mushroom species vary widely. Except for the species, the essential factors affecting trace element level in fruit bodies are the level of substrate composition, soil, pH, enzyme activity, and local pollution with trace elements. 

With this background, the aim of the study was to determine the accumulation level of minerals of three species of mushrooms collected from the Warmia and Mazury region as well as to evaluate mushroom contribution to the daily intake of the studied bioelements.

## 2. Material and Methods

**Figure 1:**
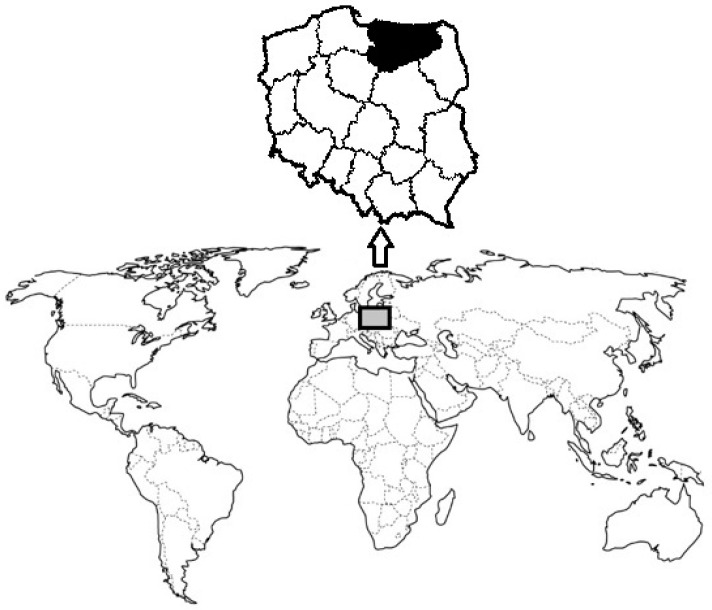
Localization of sampling of research mushroom species -Warmia and Mazury region of Poland.

The research material consisted of dried samples of king bolete (*Boletus edulis*) and bay bolete (*Boletus badius*), which were purchased from July to November from a company in the region of Warmia and Mazury. In the same period, samples of fresh chanterelles (*Cantharellus cibarius*) were obtained from mushroom selling stands, located in the province of Warmia and Mazury (Figure 1). Chanterelles were selected, cleaned (removal of impurities in the form of needles, pebbles, tree elements), and dried in a special drier according to PN-68/A-78508 [47] under laboratory conditions at 60 °C to obtain a constant weight. In total, 20 samples of each species of mushroom were collected (2 samples of each species per month), where each was divided into three-unit samples. The results in the table are presented as mean values of all samples (in total *n* = 60 per each species). In addition, each series of mineralized samples included a parallel reagent test.

### 2.1. Samples Preparation

Prepared unit samples of mushrooms were ground to a powder and weighed into glass tubes in an amount of about 1 g. The remainder was stored in closed polyethylene bags at 18 °C.

### 2.2. Samples Mineralization

Mineralization of samples was carried out according to the method described by Whiteside & Miner (1984) [48]. The weighed samples were mineralized using the "wet" method in a mixture of nitric and perchloric acids (3:1). The analysis was performed in an aluminum electric heating block with temperature programming (VELP DK 20-VELP Scientifica, Usmate, Italy), within 5–6 h, gradually increasing the temperature from 120 °C to 200 °C. The obtained colorless mineralizate was quantitatively transferred into 50 cm^3^ volumetric flasks and filled up to the mark with deionized water. Reagent samples were prepared along with the samples.

#### 2.2.1. Determination of Copper, Manganese, Iron, Zinc, Calcium and Magnesium

In previously prepared mineralizates, the contents of copper (Cu), manganese (Mn), iron (Fe), zinc (Zn), calcium (Ca), and magnesium (Mg) were determined by flame atomic absorption spectrometry (acetylene-air flame). The analyses were performed using an atomic absorption spectrometer-iCE 3000 SERIES-THERMO (Thermo-Scientific, Hemel Hempstead, Hertfordshire, UK), equipped with a GLITE data station, background correction (deuterium lamp) and appropriate cathode lamps. For the calcium determination, a 10% aqueous solution of lanthanum chloride was added to all measured solutions in a quantity ensuring a final La^+3^ concentration of 1%. The determination was carried out at wavelengths (in nm) for individual minerals: 324.8 copper; 279.5 manganese; 248.3 iron; 213.9 zinc; 285.2 magnesium; 422.7 calcium.

#### 2.2.2. Determination of Sodium and Potassium

The sodium and potassium contents (Na and K) were determined by the emission technique (acetylene-air flame). The analyzes were performed using the atomic absorption spectrometer iCE 3000 SERIES-THERMO (Thermo-Scientific, Hemel Hempstead, Hertfordshire, UK), equipped with a GLITE data station, operating in an emission system. The determination was carried out at wavelengths (in nm) for individual elements: 589.0 sodium; 766.5 potassium.

The selected concentrations of the standard solutions of individual micro- and macronutrients formed the measuring range of the analytical method used in the experiment. To determine the equation of the relationship between the measure of the signal generated by the device and the content of the analyte in the sample, calibration curves for individual elements were prepared. For this purpose, three parallel absorbance measurements were made for each standard solution, starting with measurements for the blank. Calibration curves for copper, manganese, iron, zinc, magnesium, calcium, sodium, potassium, cadmium, and lead were created based on the average absorbance values. The equations of the straight lines describing the curves were determined by the method of least squares (linear regression) according to the formula:

y = ax + b,

where a denotes slope coefficient (directional) of the straight line and b the straight line shift coefficient.

Linearity, i.e., the range of the content of the analyte, for which the output signal of the measuring device is proportional to this content, was determined based on the regression coefficient (R^2^). The value of this parameter should meet the condition R^2^ ≥ 0.999.

The accuracy of the method was checked based on an examination of the certified reference material INCT-TL-1 tea leaves, which were analyzed five times.

### 2.3. Statistical Methods

The obtained results of the content of macro- and micromineral elements in mushrooms were subjected to statistical analysis. The computer package Statistica 13.1 (StatSoft Inc., Tulsa, OK, USA) was used for calculations and MS Excel was used to present the results.

First, the measures of descriptive statistics were calculated, such as arithmetic mean, median, variance, standard deviation, and range, to characterize the distribution of values of the examined features. Then, the Kolmogorov–Smirnov test was performed to check the compliance of the distribution of the examined feature with the normal distribution and the median series test to check if the values have a random distribution.

Non-parametric tests were used to compare the average levels of feature values between samples due to a small number of samples, no normal distribution for most samples, or no random distribution of most samples.

To study the differences between the two independent groups, the U-Mann–Whitney test was used, which is equivalent to the parametric t-test for independent samples. This verifies the null hypothesis regarding “equality of means in two independent samples” against the alternative hypothesis saying that these means are not equal. When comparing average levels in several independent samples, the Kruskal–Wallis test was used to verify the null hypothesis assuming “equality of means in the tested samples” against the alternative hypothesis saying that these means are not equal. Using both tests, the significance level was *p* = 0.05. Fungi species were the assumed grouping factor. The results of the experiment and their statistical interpretation are presented in Table 1.

## 3. Results

The mean content of minerals in the studied species of mushrooms is presented in Table 1. The Cu content in the three species of mushrooms is varied. The highest amount of Cu at 48.4 mg/kg d.w. was observed in *C. cibarius*. Much lower levels were determined for *B. edulis* (23.41 mg/kg d.w.) and *B. badius* (29.7 mg/kg d.w.). Fe content turned out to be varied between species. The highest Fe content was determined for *C. cibarius* (58.9 mg/kg d.w.), followed by *B. edulis* (48.9 mg/kg d.w.). The lowest content of this element was observed in the *B. badius* (38.8 mg/kg d.w.). The content of Mn in *C. cibarius* fruit bodies (23.7 mg/kg d.w.) was significantly higher than in *B. edulis* and *B. badius* (11.3 and 11.9 mg/kg d.w., respectively). In general, *B. edulis*, *B. badius*, and *C. cibarius* differed significantly in terms of Zn content.

The highest Ca content, almost three times higher than in *B. edulis* (75.3 mg/kg d.w.) and almost four-fold than in in *B. badius* (54.7 mg/kg d.w.), was determined in *C. cibarius* and equaled 211 mg/kg d.w. The highest content of this element was determined in *B. badius* (163 mg/kg d.w.), and the lowest was in *C. cibarius* (112.7 mg/kg d.w.), while *B. edulis* contained 158 mg/kg d.w. of Zn. Significant differences in K content were found between all the analyzed fungi. The highest content of this element was determined for *C. cibarius* (46,024 mg/kg d.w.), followed by *B. badius* (36,001 mg/kg d.w.) and *B. edulis* (29,136 mg/kg d.w.). The Mg contents for *B. edulis* and *B. badius* were 566 and 526 mg/kg d.w., respectively. The content of Mg in *C. cibarius* was significantly higher than in the other mushrooms (842 mg/kg d.w.). The sodium contents appear to be varied, where more than four times lower contents than in other species (142 mg/kg d.w.) were determined in *C. cibarius*. Significant differences between the results determined for *B. edulis* (653 mg/kg d.w.) and *B. badius* (568 mg/kg d.w.) were also indicated.

Table 2 shows the calculated coverage of the daily demand for selected micro- and macroelements in the case of the consumption of 25 g of dried mushrooms, which can be equivalent to 250 g of fresh mushrooms. In relation to the recommended daily allowance (RDA) of Cu, the studied mushrooms can be a rich source of this element in the human diet. Daily demand is met to the highest degree after consumption of *C. cibarius*. It covers as much as 134–173% of the demand in children and adolescents and 173% in adults. The lowest percentage of daily demand for Cu is covered by *B. edulis*—children and adolescents: 65.0–83.6% and adults: 83.6%. The coverage of the daily requirement after consumption of *B. badius* by children and adolescents is 82.6–106% and by adults: 106%. The largest percentage of the demand for Fe in children, adolescents and adults is covered by *C. cibarius* (9.83–14.8%, 8.19–14.8%, respectively). *B. badius* covers the daily requirements to the lowest degree-among children and adolescents: 6.47–9.70% and adults: 5.39–9.70%. Values obtained for *B. edulis* are 8.15–12.2% and 6.79–12.2%, respectively. The highest daily demand for Mn is covered by *C. cibarius*: 43.3–63.5% in children and adolescents and 41.4–52.9% in adults. Comparable values were determined for *B. edulis* and *B. badius*: 12.9–18.9% (children and adolescents), 12.3–15.7% (adults), 13.6–19.9% (children and adolescents), 12.9–16.6% (adults), respectively. In the case of daily demand for Zn, the highest values were obtained for *B. badius*: children and adolescents and adults: 37.1–51.1%. Comparable amounts of recommended Zn consumption were obtained for *B. edulis*: 35.8–49.2%, while for *C. cibarius* it was 25.6–35.2%. Coverage of the daily Ca demand by consuming studied mushrooms is negligible. In the case of *B. badius* it is the lowest, 0.11% among children and adolescents and 0.11–0.14% in adults. *B. edulis* covers the recommended intake in 0.16–0.19% (adults) and 0.15 in children. Higher values were obtained for *C. cibarius*, namely 0.40% for children and adolescents, while for adults it was 0.44–0.53%. The studied mushrooms are characterized by a high K content. By consuming *C. cibarius,* the adequate intake is met in 32.9–47.9% among children and adolescents and in 32.9% in adults. The lowest degree of coverage was provided by *B. edulis* (children and adolescents: 20.8–30.4%; adults: 20.8%), while for *B. badius*, 25.7–37.5% coverage (children and adolescents) and 25.7% coverage (adults) was obtained. The demand for Mg can be realized the most after consuming *C. cibarius*. The values obtained for children and adolescents were: 5.13–8.77% and for adults: 5.01–6.79%. Recommended daily intake of Mg, in the case of *B. edulis* was 3.45–5.89% (children and adolescents) and 3.37–4.56% (adults). The values obtained for the *B. badius* were 3.21–5.48% and 3.13–4.24%, respectively. The demand for Na does not exceed 1.10% for the consumption of each of the studied species of fungi. The highest value was obtained for *B. edulis*: 1.09–1.26, then for *B. badius*: 0.95–1.09%. Daily adequate intake (AI) of Na is realized in the case of *C. cibarius* only in 0.24–0.27%.

## 4. Discussion

Brzezicha-Cirocka et al. (2016) [34] obtained similar results from research on the *C. cibarius* from Morąg (Warmia and Mazury region), where the Cu content was determined at 54 mg/kg d.w. (33–77 mg/kg d.w.). Falandysz et al. (2017) [36] also recorded a Cu content of 41 mg/kg d.w. (34–53 mg/kg d.w.) in *C. cibarius* from Poland, however in these mushrooms from China, it was 31 mg/kg d.w. Smaller amounts of Cu–31.2 mg/kg d.w. were detected by Yildiz et al. (2019) [28] in mushrooms from Turkey, while higher contents were determined by Kolundžić et al. (2017) [29] in *C. cibarius* from Serbia: 60 mg/kg d.w. Mleczek et al. (2016) [50] observed 18.7 mg/kg d.w. Cu in mushroom species growing near heavily trafficked road in Poland. Cu content in the *B. badius* ranging from 9.70–13.6 mg/kg d.w. was observed by Mleczek et al. (2013) [37], who were examining fungi from five regions of Poland. Mleczek et al. (2016) [43] analyzed species obtained from unpolluted areas of acidic sandy soils located in the Wielkopolska region and from areas where alkaline flotation tailings from copper production had been stored. They received results ranging from 14–17 mg/kg d.w. and 9–13 mg/kg d.w., respectively. These values differ from those obtained in the current study. Karmańska & Wędzisz (2010) [42] determined the average Cu content in the *B. badius* from the province of Łódź at 29.2–34.1 mg/kg d.w. while Kuziemska et al. (2019) [51] found 34.8 mg/kg d.w in Masovian Voivodeship, and Adamiak et al. (2013) [51] reported 23.4 mg/kg d.w. in this species from Wysoczyzna Siedlecka region. Karmańska & Wędzisz (2010) [42] obtained similar contents of Cu in *B. edulis*: 22.0–22.9 mg/kg d.w. Also, Brzezicha-Cirocka et al. (2016) [34] reported 15–70 and 6–72 mg/kg d.w. in *B. edulis* obtained in Morąg and on the Tarnobrzeska Plain, respectively. Liu et al. (2016) [30] determined 19–73 mg/kg d.w. of Cu in *B. edulis* from China. In this species analyzed in Africa, Rasalanavhoa et al. (2020) [27] determined 39.7–101 mg/kg d.w. of Cu. Adamiak et al. (2013) [52] obtained values of 30.6–31.8 mg/kg d.w. while examining mushrooms from the Wysoczyzna Siedlecka region (Table 3 and Table 4).

Comparable manganese contents in *C. cibarius* were obtained by Ayaz, et al. (2011) [54] 25.2 mg/kg d.w. Brzezicha-Cirocka et al. (2016) [34] determined 30 mg/kg d.w. (20–63 mg/kg d.w.) of this mineral, while Falandysz et al. (2017) [36] detected 38 mg/kg d.w. in *C. cibarius* from Poland and 19 mg/kg d.w. in this species from China. Yildiz et al. (2019) [28] determined only 4.6 mg/kg of Mn in *C. cibarius* from Turkey, while Kolundžić et al. (2017) [29] reported up to 41 mg/kg d.w. in this mushroom from Serbia (Table 5). Similar contents of Mn in *B. badius* from unpolluted areas of the Wielkopolska region, ranging from 11.3 to 14.3 mg/kg d.w. were noted by Mleczek et al. (2016) [43], as well as Karmańska & Wędzisz (2010) [42] who found 14–17 mg/kg d.w. Kojta & Falandysz (2016) [33] determined 34.5 mg/kg d.w. in *B. badius* collected from Kętrzyn and 26.5 mg/kg d.w. in this species from Augustów: Low Mn contents were determined by Mleczek et al. (2016) [43], who were examining *B. badius* samples from areas where alkaline flotation tailings from Cu production were stored: 1.4–1.8 mg/kg d.w. In the case of *B. edulis*, Brzezicha-Cirocka et al. (2016) [34] obtained results of Mn content within 9–47 mg/kg d.w. for mushrooms from Morąg and 4–15 mg/kg d.w. for this species from Tarnobrzeska Plain, where the steel mill Huta Stalowa Wola is located. Karmańska & Wędzisz (2010) [42] reported 15.5–19.2 mg/kg d.w. in *B. badius* from the province of Łódź. Rasalanavhoa et al. (2020) [27] determined 3.53–22.69 mg/kg d.w. of Mn in *B. edulis* from Africa, while Liu et al. (2016) [30] obtained values of 28–68 mg/kg d.w. in material originating from China. Karmańska & Wędzisz (2010) [42] determined the value ranging from 15.5–19.2 mg/kg d.w., while Ouzouni et al. (2007) [17] and Ayaz et al. (2011) [54] reported the value ranges of 100 to 180 mg/kg d.w.

Brzezicha-Cirocka et al. (2016) [34] determined Fe at the level of 170–520 mg/kg d.w. in *C. cibarius* from Morąg. In this species analyzed in Turkey and Serbia, 588.5 and 234 mg/kg d.w., respectively, were recorded [28,29]. Mleczek et al. (2016) [43] determined a lower Fe content in the *B. badius* in Wielkopolska region: 24–29 mg/kg d.w. and 28–35 mg/kg d.w. from a polluted area of the province of Lower Silesia. Kojta & Falandysz (2016) [33] determined the amount of this mineral at 82.5 mg/kg d.w. in the mushrooms from Kętrzyn. Mleczek et al. (2013) [37] found significantly higher contents of Fe in *B. badius* analyzing samples from five Polish provinces: 147–183 mg/kg d.w. Brzezicha-Cirocka et al. (2016) [34] received similar Fe content in *B. edulis*: 25–210 mg/kg d.w. for mushrooms from the Tarnobrzeg Plain and 51–610 mg/kg d.w. for this species obtained in Morąg. Rasalanavhoa et al. (2020) [27] determined only 20–130 mg/kg d.w. of this mineral in Africa. Much higher Fe contents in *B. badius* from the province of Łódź were found by Karmańska & Wędzisz (2010) [42]: 91.2–65.3 mg kg d.w. and Liu et al. (2016) [30] in China: 221–358 mg/kg d.w.

Similar values for Zn in *C. cibarius* from Poland were recorded by Brzezicha-Cirocka (2016) [34] (69–100 mg/kg d.w.), as well as Falandysz et al. (2017) [36] (87–100 mg/kg d.w.) and Kolundžić et al. (2017) [29] (94 mg/kg d.w.) in mushrooms from Serbia. However, Yildiz et al. (2019) [28] determined only 49.4 mg/kg d.w. of this mineral, and Ouzouni et al. (2007) [17] noted 54.1 mg/kg d.w. Adamiak et al. (2013) [52] found Zn content in *B. badius* at the level of 126 mg/kg d.w., while Mleczek et al. (2016) [43] determined quantities in the range of 72–88 mg/kg d.w. in uncontaminated areas and 86–109 mg/kg d.w. in samples from contaminated areas. Giannaccini et al. (2012) [55] and Kojta & Falandysz (2016) [33] obtained Zn content exceeding 120 mg/kg d.w., which is consistent with the data obtained as part of this study. Research carried out by Pająk (2016) [53] in the Świerklaniec Forest District, located near a metallurgical plant (Huta Zinc “Miasteczko Śląskie” [HCMŚ]), recorded a Zn level in *B. badius* at 142–305 mg/kg d.w. Similar contents of Zn were obtained by Brzezicha-Cirocka et al. (2016) [34] for *B. edulis* from Morąg: 160 mg/kg d.w. (71–220 mg/kg d.w.). These authors determined higher values for mushrooms from Tarnobrzeska Plain: 210 mg/kg d.w. (130–320 mg/kg d.w). In Africa and China, a lower content of this element was determined: 53.6–107 mg kg d.w. and 76–88 mg/kg d.w., respectively [27,30]. Further, Karmańska & Wędzisz (2010) [42] observed only 44.6–50.3 mg kg d.w. in *B. edulis* from the province of Łódź.

A comparable Mg content in *C. cibarius* was determined by Ayaz et al. (2011) [54] at the level of 815 mg/kg d.w. and Ouzouni et al. (2007) [17]: 866 mg/kg d.w. Larger amounts were observed by Brzezicha-Cirocka et al. (2016) [34]: 980–1400 mg/kg d.w. and Kolundžić et al. (2016) [29] 1426 mg/kg d.w. A significantly lower Mg content in *C. cibarius* was determined by Yildiz et al. (2019) [28] in mushrooms from Turkey: 106 mg/kg d.w. Higher Mg contents in *B. badius* were obtained by Kojta & Falandysz (2016) [33], who studied fungi from Kętrzyn (811.5 mg/kg d.w.) Comparable values were determined in *B. badius* obtained in Augustów (531.5 mg/kg d.w.) [33]. Mleczek et al. (2016) [43] observed only 82–111 mg/kg d.w. of this mineral in this species collected from unpolluted sandy soil in the Wielkopolska region. They also obtained values of 503–611 mg kg d.w. from contaminated areas. Similar contents of Mg in *B. edulis* were obtained by Liu et al. (2016) [30] in mushrooms from China (574–708 mg/kg d.w.) and Rasalanavho et al. (2020) [27] in fungi from Africa: 540–860 mg/kg d.w. Brzezicha-Cirocka et al. (2016) [34] received higher Mg contents in *B. edulis* collected in Tarnobrzeska Plain and Morąg (850–910 mg/kg d.w.) The lowest values were determined by Karmańska & Wędzisz (2010) [42] who studied mushrooms from the province of Łódź (306–324.9 mg/kg d.w.).

Generally, higher levels of Cu (670–1500 mg/kg d.w.) were obtained by Brzezicha-Cirocka et al. (2016) [34], Yildiz et al. (2019) [28] (673 mg/kg d.w.) and Kolundžić et al. (2017) [29] (92–892 mg/kg d.w.) Karmańska & Wędzisz (2010) [42] determined 154–167 mg/kg d.w. of Ca in *C. cibarius* from the provine of Łódź. In *B. badius*, there was determined a lower Ca content determined than that provided by Kojta & Falandysz (2016) [33] (197.5 mg/kg d.w.). Mleczek et al. (2016) [43] obtained the content of this mineral at the level of 24–33 mg/kg d.w. in *B. badius* collected from Polish uncontaminated acidic sandy soil and as much as 2322–2706 mg/kg d.w. from areas where alkaline flotation tailings from Cu production were stored. Karmańska & Wędzisz (2010) [42] determined 75.7–152 mg/kg d.m. of calcium in *B. badius* from the province of Łódź. Ca contents in *B. edulis* were lower than those obtained by other researchers: 110–300 mg/kg d.w. and 160–900 mg/kg d.w. [34], 384–863 mg/kg d.w. [30], and 155.5–157.8 mg/kg d.w. [42]. The most similar values were observed in Africa (26.47–206.28 mg/kg d.w.) [27].

Obtained Na contents in *C. cibarius* are lower than those given by Brzezicha-Cirocka et al. (2016) [34] who received 140–360 mg/kg of this mineral. Kolundžić et al. (2017) [29], in mushrooms originating from Serbia, determined 2431 mg/kg d.w. of Na, while Ayaz et al. (2011) [54] recorded 550 mg/kg d.w. The sodium content in the *B. badius* is comparable to that obtained by Kojta & Falandysz (2016) [33] in Kętrzyn: 519 mg/kg d.w. The amount of Na in mushrooms studied by those authors in Augustów was determined at the level of 867 mg/kg d.w. Whereas Mleczek et al. (2016) [43] received values of 226–273 mg/kg d.w. in the *B. badius* obtained from Polish uncontaminated acidic sandy soil and 121–148 mg/kg d.m. in fungi from the contaminated area. The Na results obtained for *B. edulis* were within the range specified by Liu et al. (2016) [30] (617–1184 mg/kg d.w.), Rasalanavho et al. (2020) [27] (300–1050 mg/kg d.w.) and Brzezicha-Cirocka et al. (2016) [34], who studied mushrooms from Morąg (57–1400 mg/kg d.w.). For *B. edulis* from Tarnobrzeska Plain, where the steel mill Huta Stalowa Wola is located, these authors presented lower values: 18–560 mg/kg d.w.

Similar contents of K in *C. cibarius* were found by Brzezicha-Cirocka et al. (2016) [34] (42,000–59,000 mg/kg d.w.), while much smaller amounts were determined by Kolundžić et al. (2017) [29] (18,168 mg/kg d.w.) Ayaz et al. (2011) [54] determined the values for K at 32,500 mg/kg d.w. In *B. badius*, similar K content was obtained by Kojta & Falandysz (2016) [33] in Kętrzyn at 37,500 mg/kg d.w. Smaller amounts were observed by Mleczek et al. (2016) [43] (17,584–18,932 mg/kg d.w.) in mushrooms from the province of Wielkopolska. In Cu contaminated areas, the K content was determined at 1731–1968 mg/kg d.w. [43]. In the case of *B. edulis*, a similar range of values for K was obtained by Brzezicha-Cirocka et al. (2016) [34] (27,000–32,000 mg/kg d.w.). Lower contents were determined by Rasalanavho et al. (2020) [27] in Africa: 18,180–2709 mg/kg d.w. and Liu et al. (2016) [30] (15,744–25,486 mg/kg d.w.) in China.

The results obtained in this study, regarding eight selected minerals contained in *Boletus edulis*, *Boletus badius* and *Cantharellus cibarius* from the Warmia and Mazury region do not differ much from the results presented by other authors [27,28,29,30,31,32,33,34]. However, undoubtedly, chemical composition and properties of the growing substrate, as well as the contamination of the environment determine element composition in mushrooms. The experiment confirmed the occurrence of significant differences in content of studies micro- and macroelements depending on the species of fungi. Also, the region of collecting mushrooms had an impact on the content of some minerals as there were observed differences in the values for mushrooms growing in different parts of the world (South Africa, China, Turkey, Serbia).

To understand the role of soil geochemistry and soil pollution in accumulation of minerals in mushrooms fruiting bodies several studies have been conducted in Europe [56,57,58]. A few recent ones have shown that edible mushrooms growing in unpolluted areas can accumulate Cd and Hg at much higher levels as in soil substrate, while some species hyper accumulate As, Cd, Hg, and Pb in the mining and geo-anomalous areas [22,59,60,61,62]. Research conducted by Pająk (2016) [53], who analyzed fungi collected from the Świerklaniec Forest District, located near a metallurgical plant, which is (Huta Cynku “Miasteczko Śląskie” (HCMŚ)) confirmed the high accumulation of metals, including Zn mushrooms growing in highly polluted areas, and thus the possibility of using it as a bioindicator of the degree of contamination of the natural environment with heavy metals. Mleczek et al. (2016) [43] showed the effect of substrate purity on the accumulation of individual minerals by mushroom fruiting bodies. The results revealed the existence of relationships between the content of elements and low-molecular-weight organic acids. The considerably higher content of the minerals in mushrooms growing on flotation tailings than in soil was related with higher acid contents.

## 5. Conclusions

Assuming a 100% bioavailability (in fact significantly lower as determined by many factors) of the studied minerals consumed with the analyzed mushrooms, the current study showed that these raw materials cover a significant percentage of the daily demand for many of the micro- and macroelements tested. This applies mainly to Cu, Zn, and K, although none of the species is a good source of Ca and Na. Among the mushrooms studied, *Cantharellus cibarius* is the best source of most minerals, including Cu, Fe, Mg, Ca, and K, although this requires further research to confirm the persistence of the observed trend.

The presence of minerals in human nutrition is very important. These elements are supplied to the body with food in the right proportions and determine the effectiveness of many life processes. The presence of a wide range of micro- and macroelements in edible mushrooms has prompted researchers to conduct numerous studies of the nutritional value of these raw materials. Moreover, the specificity of mushrooms and their bioindication abilities may constitute important criteria for determining their health quality and the degree of environmental pollution in their local area. The consumption of wild edible mushrooms is increasing worldwide. The knowledge and documentation of baseline mineral composition of wild growing mushrooms is essential to maintain nutritional needs, especially for many people who are vegetarian or maintain a vegan diet.

## Figures and Tables

**Table 1:** Mean content of minerals in the studied mushrooms ($\bar{x}$ ± SD), (*n* = 60) [mg/kg d.w.].

Species	Cu	Fe	Mn	Zn	Ca	K	Mg	Na
	Microelements				Macroelements			
*Boletus badius*	29.7 ± 4.22 b	38.8 ± 3.44 c	11.9 ± 2.54 b	163 ± 7.70 a	54.7 ± 7.03 b	36,001 ± 2278 b	526 ± 49.6 b	568 ± 67.3 b
*Boletus edulis*	23.4 ± 6.60 c	48.9 ± 4.00 b	11.3 ± 2.35 b	158 ± 24.1 b	75.3 ± 3.00 b	29,136 ± 1776 c	566 ± 48.0 b	653 ± 61.3 a
*Cantharellus cibarius*	48.4 ± 4.20 a	58.9 ± 5.28 a	23.7 ± 3.25 a	113 ± 9.50 c	211 ± 73.2 a	46,024 ± 4492 a	842 ± 54.2 a	142 ± 19.2 c

Explanation: values marked with the same letters do not differ significantly at significance level *p* < 0.05; $\bar{x}$—mean values, SD—standard deviation.

**Table 2:** Coverage of the daily demand for minerals after eating 25 g of dried mushrooms.

	*Boletus badius*		*Boletus edulis*		*Cantharellus cibarius*	
	Children and Youth 10–18 Years Old	Adults 19–75 Years Old	Children and Youth 10–18 Years Old	Adults 19–75 Years Old	Children and Youth 10–18 Years Old	Adults 19–75 Years Old
**Cu** (RDA)	0.70–0.90	0.90	0.70–0.90	0.90	0.70–0.90	0.90
Mean [mg/25 g]	0.74	0.74	0.59	0.59	1.21	1.21
DDC [%]	82.6–106	106	65.0–83.6	83.6	134–173	173
**Fe** (RDA)	10–15	10–18	10–15	10–18	10–15	10–18
Mean [mg/25 g]	0.97	0.97	1.22	1.22	1.48	1.48
DDC [%]	6.47–9.70	5.39–9.70	8.15–12.2	6.79–12.2	9.83–14.8	8.19–14.8
**Mn** (AI)	1.5–2.2	1.8–2.3	1.5–2.2	1.8–2.3	1.5–2.2	1.8–2.3
Mean [mg/25 g]	0.30	0.30	0.28	0.28	0.95	0.95
DDC [%]	13.6–19.9	12.9–16. 6	12.9–18.9	12.3–15.7	43.3–63.5	41.4–52.9
**Zn** (RDA)	8–11	8–11	8–11	8–11	8–11	8–11
Mean [mg/25 g]	4.09	4.09	3.94	3.94	2.82	2.82
DDC [%]	37.1–51.1	37.1–51.1	35.8–49.2	35.8–49.2	25.6–35.2	25.6–35.2
**Ca** (RDA)	1300	1000–1200	1300	1000–1200	1300	1000–1200
Mean [mg/25 g]	1.37	1.37	1.88	1.88	5.28	5.28
DDC [%]	0.11	0.11–0.14	0.15	0.16–0.19	0.40	0.44–0.53
**K** (AI)	2400–3500	3500	2400–3500	3500	2400–3500	3500
Mean [mg/25 g]	900	900	728	3728	1150	1150
DDC [%]	25.7–37.5	25.7	20.8–30.4	20.8	32.9–47.9	32.9
**Mg** RDA)	240–410	310–420	240–410	310–420	240–410	310–420
Mean [mg/25 g]	13.2	13.2	14.1	14.1	21.1	21.1
DDC [%]	3.21–5.48	3.13–4.24	3.45–5.89	3.37–4.56	5.13–8.77	5.01–6.79
**Na** (AI)	1300–1500	1300–1500	1300–1500	1300–1500	1300–1500	1300–1500
Mean [mg/25 g]	14.2	14.2	16.3	16.3	3.56	3.56
DDC [%]	0.95–1.09	0.95–1.09	1.09–1.26	1.09–1.26	0.24–0.27	0.24–0.27

Source: own study based on Jarosz et al. 2017 [49]. Explanation: RDA—Recommended Daily Allowance [mg/person]; AI—Adequate Intake [mg/person]; DDC—Daily demand coverage.

**Table 3:** Minerals in *Boletus edulis* (adapted) [mg/kg d.w.].

	Cu	Fe	Mn	Zn	Ca	K	Mg	Na	Loc.	Ref.
	Microelements				Macroelements					
Mean Range	nd 19–73	nd 221–358	nd 28–69	nd 76–88	nd 384–863	nd 15,744–25,486	nd 574–708	nd 617–1184	Southwest China	[30]
Mean Range	37 15–70	200 51–610	21 9–47	160 71–220	480 160–900	27,000 21,000–31,000	910 680–1300	360 57–1400	Morąg, Poland	[34]
Mean Range	36 6–72	47 25–210	8,6 4–15	210 130–320	200 110–300	32,000 24,000–41,000	850 680–1000	190 18–560	Tarnobrzeska Plain, Poland	[34]
Mean Range	nd 39.7–101	nd 20–130	nd 3.53–22.7	nd 53.6–107	nd 26.5–206	nd 18,180–27,090	nd 540–860	nd 300–1050	KwaZulu–Natal, South Africa	[27]
Mean Range	nd 22.0–22.9	nd 65.3–91.2	nd 15.5–19.2	nd 44.6–50.3	nd 155–158	nd nd	nd 306–325	nd nd	The province of Łódź, Poland	[42]
Mean Range	30.9 30–31.8	nd nd	nd nd	137 131–140	nd nd	nd nd	nd nd	nd nd	Wysoczyzna Siedlecka, Poland	[52]

Explanation: Loc.—lokalization; Ref.—references; nd—no data.

**Table 4:** Minerals in *Boletus badius* (adapted) [mg/kg d.w.].

	Cu	Fe	Mn	Zn	Ca	K	Mg	Na	Loc.	Ref.
	Microelements				Macroelements					
Mean Range	nd nd	82.5 49.5–107	34.5 16–57	252 123–409	197 85–365	37,500 31,000–44,500	811.5 676–1042	519 315–719	Kętrzyn, Poland	[33]
Mean Range	nd nd	nd nd	26.5 18.0–34.5	138 116–159	nd nd	30,500 26,000–35,500	531.5 481–644	867 672–1636	Augustów, Poland	[33]
Mean Range	nd 9.70–13.6	nd 147–183	nd nd	nd nd	nd nd	nd nd	nd nd	nd nd	Pomeranian Greater Poland, Łódź, Lower Silesian	[37]
Mean Range	nd 14–17	nd 24–29	nd 11.3–14.3	nd 72–88	nd 24–33	nd 17,584–18,932	nd 82–111	nd 226–273	Wielkopolska region, Poland	[43]
Mean Range	nd 9–13	nd 28–35	nd 1.4–1.8	nd 86–109	nd 2322–2706	nd 1731–1968	nd 503–611	nd 121–148	The Lower Silesia region, Poland	[43]
Mean Range	nd 29.2–34.1	nd 52.0–73.1	nd 14–17	nd 43.3–46.9	nd 75.7–152	nd nd	nd 265–293	nd nd	The province of Łódź, Poland	[42]
Mean Range	23.4 22.3–25.4	nd nd	nd nd	126 121–131	nd nd	nd nd	nd nd	nd nd	Wysoczyzna Siedlecka, Poland	[52]
Mean Range	nd nd	nd nd	nd nd	nd 148–305	nd nd	nd nd	nd nd	nd nd	Nadleśnictwo Świerklaniec, Poland	[53]

Explanation: Loc.—lokalization; Ref.—references; nd—no data.

**Table 5:** Minerals in *Cantharellus cibarius* (adapted) [mg/kg d.w.].

	Cu	Fe	Mn	Zn	Ca	K	Mg	Na	Loc.	Ref.
	Microelements				Macroelements					
Mean Range	54 33–77	330 170–520	30 20–63	82 69–100	1000 670–1500	49,000 42,000–59,000	1200 980–1400	240 140–360	Morąg, Poland	[34]
Mean Range	41.0 34–53	nd nd	38 19–51	92 87–100	nd nd	nd nd	nd nd	nd nd	Poland	[36]
Mean Range	31 nd	nd nd	19 nd	76 nd	nd nd	nd nd	nd nd	nd nd	Yuxi, China	[36]
Mean Range	31.2 nd	588 nd	4.60 nd	49.4 nd	673 nd	nd nd	nd nd	nd nd	Kastamonu, Turkey	[28]
Mean Range	60 nd	234 nd	41 nd	94 nd	92,892 nd	18,168 nd	1426 nd	2431 nd	Soko Banja, Serbia	[29]
Mean Range	nd 34.8–38.5	nd 89.4–91.7	nd 42.7–45.6	nd 43.1–45.1	nd 154–167	nd nd	nd 305–376	nd nd	The province of Łódź, Poland	[42]
Mean Range	37.3 nd	130 nd	25.2 nd	nd nd	722 nd	32,500 nd	815 nd	550 nd	East Black Sea, Turkey	[54]
Mean Range	32.6 nd	119 nd	22.1 nd	54.1 nd	nd nd	nd nd	866 nd	nd nd	Epirus, West Macedonia, Greece	[17]

Explanation: Loc.—lokalization; Ref.—references; nd—no data.
